# Missing data strategies for the Patient-Reported Outcomes version of the Common Terminology Criteria for Adverse Events (PRO-CTCAE) in Alliance A091105 and COMET-2

**DOI:** 10.1007/s11136-021-02968-1

**Published:** 2021-08-21

**Authors:** Gina L. Mazza, Molly M. Petersen, Brenda Ginos, Blake T. Langlais, Narre Heon, Mrinal M. Gounder, Michelle R. Mahoney, Alexander J. Zoroufy, Gary K. Schwartz, Lauren J. Rogak, Gita Thanarajasingam, Ethan Basch, Amylou C. Dueck

**Affiliations:** 1. Alliance Statistics and Data Center, Mayo Clinic, Scottsdale, AZ, USA; 2. Department of Quantitative Health Sciences, Mayo Clinic, Scottsdale, AZ, USA; 3. Memorial Sloan Kettering Cancer Center, New York, NY, USA; 4. IQVIA Institute for Human Data Science, Raleigh-Durham, NC, USA; 5. Alliance Statistics and Data Center, Mayo Clinic, Rochester, MN, USA; 6. Department of Quantitative Health Sciences, Mayo Clinic, Rochester, MN, USA; 7. Herbert Irving Comprehensive Cancer Center, Columbia University, New York, NY, USA; 8. Division of Hematology, Mayo Clinic, Rochester, MN, USA; 9. UNC Lineberger Comprehensive Cancer Center, University of North Carolina, Chapel Hill, NC, USA

## Abstract

**Purpose:**

Missing scores complicate analysis of the Patient-Reported Outcomes version of the Common Terminology Criteria for Adverse Events (PRO-CTCAE) because patients with and without missing scores may systematically differ. We focus on optimal analysis methods for incomplete PRO-CTCAE items, with application to 2 randomized, double-blind, placebo-controlled, phase III trials.

**Methods:**

In Alliance A091105 and COMET-2, patients completed PRO-CTCAE items before randomization and several times post-randomization (*N* = 64 and 107, respectively). For each trial, we conducted between-arm comparisons on the PRO-CTCAE via complete-case two-sample *t*-tests, mixed modeling with contrast, and multiple imputation followed by two-sample *t*-tests. Because interest lies in whether CTCAE grades can inform missing PRO-CTCAE scores, we performed multiple imputation with and without CTCAE grades as auxiliary variables to assess the added benefit of including them in the imputation model relative to only including PRO-CTCAE scores across all cycles.

**Results:**

PRO-CTCAE completion rates ranged from 100.0% to 71.4% and 100.0% to 77.1% across time in A091105 and COMET-2, respectively. In both trials, mixed modeling and multiple imputation provided the most similar estimates of the average treatment effects. Including CTCAE grades in the imputation model did not consistently narrow confidence intervals of the average treatment effects because correlations for the same PRO-CTCAE item between different cycles were generally stronger than correlations between each PRO-CTCAE item and its corresponding CTCAE grade at the same cycle.

**Conclusion:**

For between-arm comparisons, mixed modeling and multiple imputation are informative techniques for handling missing PRO-CTCAE scores. CTCAE grades do not provide added benefit for informing missing PRO-CTCAE scores.

## Introduction

To incorporate the patient perspective into assessments of symptomatic adverse events, the National Cancer Institute contracted development of the Patient-Reported Outcomes version of the Common Terminology Criteria for Adverse Events (PRO-CTCAE) [1]. The PRO-CTCAE is a library of 124 items assessing the frequency, severity, interference, amount, and/or presence of 78 symptomatic adverse events drawn from the CTCAE (http://healthcaredelivery.cancer.gov/pro-ctcae). Patients complete a subset of items based on symptomatic adverse events most relevant to the treatment(s) under investigation. The National Cancer Institute recommends reporting patients’ PRO-CTCAE scores in conjunction with clinicians’ CTCAE grades to improve the evaluation of symptomatic adverse events in cancer clinical trials [2]. Relative to clinician reports, patient reports show greater sensitivity to changes in daily functioning or symptom burden, thus allowing for improvements in safety monitoring, symptom management, and even survival [3, 4]. Furthermore, the PRO-CTCAE may be better able to capture when treatments produce less severe but more chronically bothersome adverse events relative to the CTCAE (where grades 4 and 5 correspond to life-threatening adverse events and death, respectively), and thereby enable better understanding of treatment tolerability from the patient perspective.

However, missing scores complicate reporting and analysis of the PRO-CTCAE. Patients with and without missing scores may systematically differ, thus jeopardizing the validity and generalizability of clinical trial results. For example, patients with more severe symptomatic adverse events might drop out or feel too unwell to complete PRO-CTCAE assessments, such that these patients miss PRO-CTCAE assessments more often than patients with less severe symptomatic adverse events. Analyses based on only the remaining patients’ PRO-CTCAE scores may result in biased parameter estimates and invalid conclusions.

This paper focuses on optimal analysis methods for incomplete PRO-CTCAE items (Alliance A151912), with application to 2 randomized, double-blind, placebo-controlled, phase III trials: Alliance A091105 and COMET-2. We conduct between-arm comparisons within each trial while comparing the following strategies for addressing missing PRO-CTCAE scores: complete-case two-sample *t*-test, mixed modeling with contrast, and multiple imputation followed by a two-sample *t*-test. Because interest lies in whether CTCAE grades can inform missing PRO-CTCAE scores, we then perform multiple imputation with and without CTCAE grades as auxiliary variables to assess the added benefit of including CTCAE grades in the imputation model relative to only including PRO-CTCAE scores across all cycles.

## Methods

### Patients, Measures, and Procedures

In A091105, patients with desmoid tumors or deep fibromatosis were randomized 2:1 to receive sorafenib or placebo by mouth once daily. Upon confirmation of progression, patients assigned to the placebo arm were allowed to cross over to open-label use of sorafenib. The primary endpoint was progression-free survival (NCT02066181; see Gounder et al. [5] for results). English-speaking patients were invited to participate in a correlative study of patient-reported pain, symptomatic adverse events, and quality of life while on randomized treatment. Patients enrolled in the correlative study completed 19 PRO-CTCAE items via paper booklets prior to randomization and after each 4-week cycle for 8 cycles while on randomized treatment (i.e., Weeks 4, 8, 12, 16, 20, 24, 28, and 32). The PRO-CTCAE items assessed the frequency (F), severity (S), interference (I), and/or presence (P) of the following symptoms: insomnia (SI), constipation (S), pain (FSI), fatigue (SI), nausea (FS), vomiting (FS), diarrhea (F), rash (P), hand-foot syndrome (SI),^1^ decreased appetite (SI), and mouth or throat sores (S). Clinicians graded patients’ fatigue, papulopustular rash, palmar-plantar erythrodysesthesia syndrome, diarrhea, anorexia, nausea, vomiting, abdominal pain, mucositis oral, hypertension, arthralgia, and myalgia via the CTCAE v4.0 at each cycle. For adverse events beyond those solicited at each cycle, clinicians reported grade 1 and 2 adverse events with attributions of possible, probable, or definite and all grade 3+ adverse events regardless of attribution. The institutional review board or ethics committee at each participating site approved the protocol, and patients provided written informed consent.

In COMET-2, patients with metastatic castration-resistant prostate cancer and narcotic-dependent pain from bone metastases who had progressed after treatment with docetaxel and either abiraterone or enzalutamide were randomized 1:1 to receive cabozantinib by mouth once daily or mitoxantrone every 3 weeks plus prednisone by mouth twice daily (with matching placebos in each arm). The primary endpoint was pain response at Week 6 confirmed at Week 12 (NCT01522443; see Basch et al. [6] for results). Patients completed 21 PRO-CTCAE items via an interactive voice response system prior to randomization; at Weeks 3, 6, and 12; and every 6 weeks thereafter until progression. The PRO-CTCAE items assessed the frequency (F), severity (S), interference (I), and/or presence (P) of the following symptoms: insomnia (SI), constipation (S), pain (FSI), fatigue (SI), nausea (FS), vomiting (FS), diarrhea (F), rash (P), decreased appetite (SI), numbness or tingling in hands or feet (SI), mouth or throat sores (S), and shortness of breath (SI). Clinicians graded patients’ adverse events via the CTCAE v4.0 while recording start/stop dates for each adverse event. The institutional review board or ethics committee at each participating site approved the protocol, and patients provided written informed consent.

### Statistical Analysis

All analyses were performed separately for A091105 and COMET-2. As recommended by the Setting International Standards in Analyzing Patient-Reported Outcomes and Quality of Life Endpoints Data (SISAQOL) Consortium [7], the PRO-CTCAE completion rate and available data rate were calculated at each cycle. The available data rate was calculated as the ratio of the number of patients with observed PRO-CTCAE scores to the total number of patients. The completion rate was calculated as the ratio of the number of patients with observed PRO-CTCAE scores to the number of patients eligible to complete the PRO-CTCAE assessment at that time point. That is, the completion rate’s denominator excluded patients who were no longer required to complete the PRO-CTCAE assessment per the protocol (e.g., due to death or going off randomized treatment). In A091105, reported reasons for missing PRO-CTCAE scores were summarized at each cycle.

Between-arm comparisons on the PRO-CTCAE at Week 12^2^ were conducted using the following strategies: complete-case two-sample *t*-test, mixed modeling with contrast, and multiple imputation followed by a two-sample *t*-test. The complete-case two-sample *t*-test assumes a missing completely at random mechanism, meaning the probability of missingness is unrelated to the observed and missing scores. Excluding patients with missing scores yields unbiased parameter estimates under a missing completely at random mechanism because the observed scores can be regarded as a random subsample of the hypothetical complete scores. Mixed modeling and multiple imputation assume a missing at random mechanism, meaning the probability of missingness is unrelated to the missing scores after conditioning on the observed scores. The missing at random mechanism is a much more plausible assumption than the missing completely at random mechanism. Mixed modeling uses maximum likelihood estimation and includes patients’ responses to a PRO-CTCAE item at all available cycles, such that patients with *any* observed scores contribute to the mixed model. In doing so, mixed modeling accounts for missing PRO-CTCAE scores using patients’ responses to the same PRO-CTCAE item at different cycles (but not patients’ responses to other PRO-CTCAE items or clinicians’ CTCAE grades). Multiple imputation involves creating multiple copies of the data set with different imputed scores (imputation phase), analyzing the imputed data sets as though they were complete data sets (analysis phase), and pooling the parameter estimates and standard errors across the imputed data sets to yield a single set of results (pooling phase). Multiple imputation quantifies uncertainty due to the missing scores and inflates the standard errors accordingly based on changes in the imputed scores across the imputed data sets.

Multiple imputation was performed with and without CTCAE grades as auxiliary variables to assess the added benefit of including CTCAE grades in the imputation model relative to only including PRO-CTCAE scores across all cycles. To impute the missing scores, multiple imputation uses available information about patients’ would-be responses to the incomplete PRO-CTCAE items. This information may include patients’ responses to the same PRO-CTCAE item at different cycles, patients’ responses to other PRO-CTCAE items, and/or clinicians’ CTCAE grades (i.e., so-called auxiliary variables). For example, a patient who reports no pain at Cycle 2 may be more likely to report no pain at Cycle 3. A patient who reports severe insomnia at Cycle 3 may be more likely to report severe fatigue at Cycle 3. If a clinician reports grade 1+ vomiting at Cycle 3, then the patient may be more likely to report severe vomiting at Cycle 3. This information helps multiple imputation make plausible guesses about what patients’ missing scores would have been had they completed the PRO-CTCAE assessment. In the imputation model, auxiliary variables correlate with an incomplete PRO-CTCAE item and/or missingness. Auxiliary variables that correlate with an incomplete PRO-CTCAE item improve power by providing information about the missing scores; auxiliary variables that correlate with an incomplete PRO-CTCAE item and its missingness indicator (0 = missing, 1 = observed) reduce bias [8]. In general, the imputation model should include auxiliary variables that correlate at ≥ 0.40 (in magnitude) with both an incomplete PRO-CTCAE item and its missingness indicator or correlate at ≥ 0.50 (in magnitude) with an incomplete PRO-CTCAE item [8–11]. Auxiliary variables generally should not have more than 10% of their scores concurrently missing with the incomplete PRO-CTCAE item [9, 12]. For example, in A091105, most patients who did not complete the PRO-CTCAE item on insomnia severity at Cycle 3 also did not complete the PRO-CTCAE item on fatigue severity at Cycle 3 due to missing the PRO-CTCAE assessment altogether. Thus, our imputation model only included patients’ responses to the same PRO-CTCAE item at different cycles and clinicians’ CTCAE grades as auxiliary variables.

All analyses were performed in SAS 9.4 (SAS Institute Inc., Cary, NC). The complete-case two-sample *t*-test was conducted using the TTEST procedure (for PRO-CTCAE items assessing frequency, severity, or interference); complete-case logistic regression was conducted using the LOGISTIC procedure (for the PRO-CTCAE item assessing presence). Mixed modeling was performed using the MIXED procedure (for PRO-CTCAE items assessing frequency, severity, or interference) or GLIMMIX procedure (for the PRO-CTCAE item assessing presence). Each mixed model included a fixed intercept; fixed effects for time, arm, and arm by time interaction; and autoregressive (lag 1) residual covariance matrix that accounts for repeated PRO-CTCAE assessments within patients. Time was treated as nominal, such that the mixed models did not make assumptions about the trajectory of patients’ PRO-CTCAE scores over time. A logit link function was specified within the mixed model for the PRO-CTCAE item assessing presence. Multiple imputation was conducted twice for each PRO-CTCAE item using the MI procedure. In the first imputation model, each PRO-CTCAE item was imputed based on patients’ responses to that PRO-CTCAE item at all available cycles. In the second imputation model, each PRO-CTCAE item was imputed based on patients’ responses to that PRO-CTCAE item at all available cycles plus relevant CTCAE grades at Week 12 (Table 1). Because clinician-reported adverse events were collected by start/stop dates in COMET-2, CTCAE grades for ongoing adverse events at Week 12 (±1 week) were used as auxiliary variables. For example, in A091105, the PRO-CTCAE item on hand-foot syndrome severity was imputed based on patients’ responses to that PRO-CTCAE item at all available cycles plus clinicians’ CTCAE grades for palmar-plantar erythrodysesthesia syndrome at Week 12. Similarly, in COMET-2, the PRO-CTCAE item on numbness or tingling in hands or feet was imputed based on patients’ responses to that PRO-CTCAE item at all available cycles plus clinicians’ CTCAE grades for peripheral neuropathy at Week 12. Using a fully conditional specification, 50 imputed data sets were generated while setting the number of burn-in iterations (i.e., the number of iterations prior to saving each imputed data set) to 1,000. A two-sample *t*-test was performed on each imputed data set using the REG procedure (for PRO-CTCAE items assessing frequency, severity, or interference) or LOGISTIC procedure (for the PRO-CTCAE item assessing presence), and results were pooled across the imputed data sets using the MIANALYZE procedure.

To assess similarity of results, estimates of average treatment effects at Week 12 and statistical efficiency were compared across strategies for addressing missing PRO-CTCAE scores. Statistical efficiency was measured by confidence interval width, with narrower confidence intervals indicating greater statistical efficiency.

## Results

### Patient Demographic and Disease Characteristics

Of the 87 patients enrolled in A091105, 64 consented to participate in the correlative study. Of these 64 patients, 36 (56.3%) were randomized to the sorafenib arm and 28 (43.8%) were randomized to the placebo arm. The randomization ratio was not 2:1 (sorafenib:placebo) as specified in the protocol due to a randomization algorithm error that was detected and corrected partway through enrollment [5]. Patients were 65.6% female. Median age was 36.5 years (range = 18 to 68 years). Baseline ECOG performance status was 0 for 39 (60.9%) patients and 1 for 25 (39.1%) patients. Intraabdominal disease was present in 21 (32.8%) patients. Disease status was newly diagnosed for 32 (50.0%) patients, recurrent for 30 (46.9%) patients, and not reported for 2 (3.1%) patients. Patients were unblinded on November 17, 2017, and those receiving placebo were allowed to cross over to open-label use of sorafenib if progression had not yet occurred.

Of the 119 patients enrolled in COMET-2, 107 completed the baseline PRO-CTCAE assessment and at least 1 post-baseline PRO-CTCAE assessment and were thus included in this analysis. Of these 107 patients, 53 (49.5%) were randomized to the cabozantinib arm and 54 (50.5%) were randomized to the mitoxantrone-prednisone arm. Median age was 65 years (range = 44 to 80 years). Baseline ECOG performance status was 0 or 1 for 94 (87.9%) patients. All patients had undergone at least 2 prior lines of systemic treatment for castration-resistant metastatic prostate cancer.

### PRO-CTCAE Completion

In A091105, 100.0% to 52.8% and 100.0% to 39.3% of those randomized to the sorafenib and placebo arms, respectively, completed the PRO-CTCAE assessment between baseline and Week 32 (Table 2). When excluding patients who were no longer required to complete the PRO-CTCAE assessment per the protocol, completion rates ranged from 100.0% to 70.4% and 100.0% to 73.3%, respectively (Table 2). At Week 12, 13 patients did not complete the PRO-CTCAE assessment due to being off randomized treatment (*n* = 7), not having a clinic visit (*n* = 2), staff not administering the questionnaire booklet (*n* = 2), or unspecified reason (*n* = 2). Of the 51 patients who completed the PRO-CTCAE assessment at Week 12, 50 completed all 19 items and 1 missed 5 of the 19 items due to skipping a page of the questionnaire booklet.

In COMET-2, 100.0% to 28.3% and 100.0% to 22.2% of those randomized to the cabozantinib and mitoxantrone-prednisone arms, respectively, completed the PRO-CTCAE assessment between baseline and Week 24 (Table 2). When excluding patients who were no longer required to complete the PRO-CTCAE assessment per the protocol, completion rates ranged from 100.0% to 68.2% and 100.0% to 81.0%, respectively (Table 2).

### Comparison of Missing Data Strategies

Tables 3 and 4 summarize between-arm comparisons on the PRO-CTCAE at Week 12 based on a complete-case two-sample *t*-test, mixed modeling with contrast, and multiple imputation followed by a two-sample *t*-test in A091105 and COMET-2, respectively. In both trials, mixed modeling and multiple imputation provided the most similar estimates of the average treatment effect at Week 12 for PRO-CTCAE items assessing frequency, severity, or interference (Tables 3 and 4). In A091105, differences between these estimates ranged from −0.313 to 0.131 when comparing the complete-case two-sample *t*-test and mixed modeling, −0.265 to 0.103 when comparing the complete-case two-sample *t*-test and multiple imputation, and −0.060 to 0.068 when comparing mixed modeling and multiple imputation. In COMET-2, differences between these estimates ranged from −0.158 to 0.132 when comparing the complete-case two-sample *t*-test and mixed modeling, −0.194 to 0.174 when comparing the complete-case two-sample *t*-test and multiple imputation, and −0.065 to 0.085 when comparing mixed modeling and multiple imputation.

In A091105, the sample size used for analysis was consistently 64 (i.e., all patients enrolled who consented to participate in the correlative study) for mixed modeling and multiple imputation, whereas the sample size used for analysis was 50 or 51 (i.e., patients who consented to participate in the correlative study and completed the relevant PRO-CTCAE item at Week 12) for the complete-case two-sample *t*-test (Table 3). Mixed modeling yielded confidence intervals that were 96.5% as wide as those generated by a complete-case two-sample *t*-test, multiple imputation yielded confidence intervals that were 106.3% as wide as those generated by a complete-case two-sample *t*-test, and mixed modeling yielded confidence intervals that were 91.2% as wide as those generated by multiple imputation, on average, for PRO-CTCAE items assessing frequency, severity, or interference. In COMET-2, the sample size used for analysis was consistently 107 for mixed modeling and multiple imputation, whereas the sample size used for analysis was 75 or 76 for the complete-case two-sample *t*-test (Table 4). Mixed modeling yielded confidence intervals that were 87.8% as wide as those generated by a complete-case two-sample *t*-test, multiple imputation yielded confidence intervals that were 99.1% as wide as those generated by a complete-case two-sample *t*-test, and mixed modeling yielded confidence intervals that were 88.7% as wide as those generated by multiple imputation, on average, for PRO-CTCAE items assessing frequency, severity, or interference.

### CTCAE Grades as Auxiliary Variables

Table 5 provides CTCAE grades for A091105 and COMET-2 at Week 12. Notably, the proportion of nonzero CTCAE grades in A091105 was very low for several clinician-reported adverse events. In A091105, correlations between the PRO-CTCAE items and corresponding CTCAE grades at Week 12 varied widely (range = 0.014 to 0.627; Table 1). The strongest correlation occurred between patient-reported severity of mouth or throat sores and clinician-reported mucositis oral (*r* = 0.627), though this correlation was inflated due to observing very few nonzero CTCAE grades for mucositis oral at Week 12 (i.e., 3/64, 4.7%; Table 5). Other strong correlations occurred between patient-reported severity of hand-foot syndrome and clinician-reported palmar-plantar erythrodysesthesia syndrome (*r* = 0.487), patient-reported frequency of nausea and clinician-reported nausea (*r* = 0.447), patient-reported severity of vomiting and clinician-reported vomiting (*r* = 0.441), and patient-reported interference of fatigue and clinician-reported fatigue (*r* = 0.436). The weakest correlations occurred between patient-reported interference of pain and clinician-reported arthralgia (*r* = 0.014), patient-reported severity of pain and clinician-reported myalgia (*r* = 0.059), and patient-reported frequency of diarrhea and clinician-reported diarrhea (*r* = 0.096).

Relative to A091105, COMET-2 had a much higher proportion of nonzero CTCAE grades due to targeting a more advanced cancer patient population and administering a more toxic chemotherapy regimen (Table 5). In COMET-2, correlations between the PRO-CTCAE items and corresponding CTCAE grades at Week 12 varied widely (range = −0.102 to 0.445; Table 1). The strongest correlations occurred between patient-reported frequency and severity of vomiting and clinician-reported vomiting (*r* = 0.427 and 0.445, respectively). Clinician-reported insomnia, fatigue, decreased appetite, and peripheral neuropathy did not strongly correlate with their patient-reported counterparts (range = −0.102 to 0.046; Table 1).

In both trials, between-arm comparisons on the PRO-CTCAE at Week 12 were similar regardless of whether CTCAE grades were included in the imputation model (Tables 3 and 4). Including clinicians’ CTCAE grades did not consistently narrow the confidence intervals associated with these average treatment effects (Tables 3 and 4). In A091105, on average, multiple imputation with auxiliary variables yielded confidence intervals that were 98.0% as wide as those generated by multiple imputation without auxiliary variables. When included in the imputation model, the CTCAE grades with the weakest correlations with the PRO-CTCAE scores widened the confidence intervals (maximum = 106.7% as wide as the confidence interval generated by multiple imputation without auxiliary variables). Similarly, in COMET-2, on average, multiple imputation with auxiliary variables yielded confidence intervals that were 101.3% as wide as those generated by multiple imputation without auxiliary variables. These results are consistent with the correlation patterns observed among the PRO-CTCAE items and CTCAE grades in this sample. That is, correlations for the same PRO-CTCAE item between different cycles were generally stronger than correlations between each PRO-CTCAE item and its corresponding CTCAE grade at the same cycle. These results are also consistent with the data sparseness observed for several clinician-reported adverse events. That is, in A091105, most CTCAE grades equaled 0 or 1 at Week 12 (Table 5).

## Conclusion

Properly handling missing PRO-CTCAE scores supports more accurate and generalizable causal inferences regarding treatment tolerability. In this paper, we conducted between-arm comparisons while applying the following strategies for addressing missing PRO-CTCAE scores in A091105 and COMET-2: complete-case two-sample *t*-test, mixed modeling with contrast, and multiple imputation followed by a two-sample *t*-test. In both trials, mixed modeling and multiple imputation provided the most similar estimates of the average treatment effect. These results are unsurprising because, unlike a complete-case two-sample *t*-test, mixed modeling and multiple imputation provide unbiased parameter estimates under a missing at random mechanism and do not exclude patients with missing scores—highly desirable features per the SISAQOL Consortium [7].

We also performed multiple imputation with and without CTCAE grades as auxiliary variables to assess the added benefit of including CTCAE grades in the imputation model relative to only including PRO-CTCAE scores across all cycles. Our results suggest that CTCAE grades can inform missing PRO-CTCAE scores for any adverse events that show strong agreement between clinician and patient reports, though model simplicity and computational ease may warrant use of other strategies. In A091105 and COMET-2, inclusion of CTCAE grades in the imputation model was not worthwhile because the strongest correlations occurred among the same PRO-CTCAE item at different cycles. These results make sense because the information provided by patients often differs from the information provided by clinicians. Thus, we recommend using patients’ PRO-CTCAE scores for the same symptom at different cycles to inform patients’ missing PRO-CTCAE scores. This can be accomplished via mixed modeling or multiple imputation. Although multiple imputation is widely available in statistical software packages, multiple imputation is much more demanding procedurally than mixed modeling. The user must examine convergence diagnostics; ensure the imputation model includes all the analysis variables; create, manage, and analyze multiple imputed data sets; and pool the results. The extent to which statistical software packages automate this procedure varies. Conducting mixed modeling for PRO-CTCAE items assessing frequency, severity, or interference is consistent with recommendations outlined by the SISAQOL Consortium [7]. However, multiple imputation may outperform mixed modeling for PRO-CTCAE items assessing presence as mixed modeling can result in convergence issues with binary endpoints.

Limitations of this work include our focus on 2 phase III trials with modest sample sizes. Examining other phase II and phase III trials as well as other patient populations may improve the generalizability of these results. However, the A091105 and COMET-2 sample sizes are not atypical for plausible applications of these strategies for addressing missing PRO-CTCAE scores. Another limitation is our focus on the similarity of parameter estimates and differences in statistical efficiency across the complete-case two-sample *t*-test, mixed modeling with contrast, and multiple imputation followed by a two-sample *t*-test. Because we did not simulate the data, we cannot calculate bias of the parameter estimates. However, our results can serve as the basis for a future simulation study evaluating strategies for addressing missing PRO-CTCAE scores.

In summary, using patients’ PRO-CTCAE scores for the same symptom at different cycles to inform patients’ missing PRO-CTCAE scores can mitigate problems associated with missing scores.

Accurately evaluating patients’ PRO-CTCAE scores promotes the safety and tolerability of treatments as well as improves the implementation and interpretation of cancer clinical trials.

## Figures and Tables

**Table 1.** CTCAE Grades Used as Auxiliary Variables in the Imputation Models

PRO-CTCAE Item	A091105		COMET-2	

	CTCAE Grades	Week 12 Correlation	CTCAE Grades	Week 12 Correlation
Insomnia (S)	—	—	Insomnia	0.034
Insomnia (I)	—	—	Insomnia	−0.027
Constipation (S)	—	—	Constipation	0.320
Pain (F)	Abdominal Pain, Arthralgia, Myalgia	0.205, 0.242, 0.100	Back Pain, Pain in Extremity, Arthralgia	0.168, 0.140, 0.312
Pain (S)	Abdominal Pain, Arthralgia, Myalgia	0.151, 0.141, 0.059	Back Pain, Pain in Extremity, Arthralgia	0.127, 0.088, 0.133
Pain (I)	Abdominal Pain, Arthralgia, Myalgia	0.142, 0.014, 0.167	Back Pain, Pain in Extremity, Arthralgia	0.109, 0.111, 0.222
Fatigue (S)	Fatigue	0.360	Fatigue	−0.070
Fatigue (I)	Fatigue	0.436	Fatigue	−0.102
Nausea (F)	Nausea	0.447	Nausea	0.209
Nausea (S)	Nausea	0.325	Nausea	0.112
Vomiting (F)	Vomiting	0.246	Vomiting	0.427
Vomiting (S)	Vomiting	0.441	Vomiting	0.445
Diarrhea (F)	Diarrhea	0.096	Diarrhea	0.361
Rash (P)	Papulopustular Rash	0.302	Rash	−0.064
Hand-Foot Syndrome (S)	Palmar-Plantar Erythrodysesthesia Syndrome	0.487		
Hand-Foot Syndrome (I)	Palmar-Plantar Erythrodysesthesia Syndrome	0.395		
Decreased Appetite (S)	Anorexia	0.374	Decreased Appetite	−0.068
Decreased Appetite (I)	Anorexia	0.359	Decreased Appetite	−0.056
Mouth/Throat Sores (S)	Mucositis Oral	0.627	Mucositis Oral	0.307
Numbness/Tingling (S)			Peripheral Neuropathy	0.225
Numbness/Tingling (I)			Peripheral Neuropathy	0.046
Shortness of Breath (S)			Dyspnea	0.367
Shortness of Breath (I)			Dyspnea	0.315

Gray shading indicates that the PRO-CTCAE item was not administered in that trial. The column of correlations provides correlations between each patient-reported adverse event at Week 12 and its corresponding clinician-reported adverse event at Week 12. In A091105, *N* = 51 for all PRO-CTCAE items at Week 12 except frequency and severity of pain (*N* = 50). In COMET-2, *N* = 76 for all PRO-CTCAE items at Week 12 except severity and interference of shortness of breath (*N* = 75). PRO-CTCAE: Patient-Reported Outcomes version of the Common Terminology Criteria for Adverse Events; CTCAE: Common Terminology Criteria for Adverse Events; (F): frequency; (S): severity; (I): interference; (P): presence.

**Table 2.** PRO-CTCAE Completion Rates and Available Data Rates

Time Point	Completion Rate (# Completed / # Eligible for Completion)		Available Data Rate (# Completed / # Consented)	
	A091105			
	Sorafenib	Placebo	Sorafenib	Placebo
Baseline	36 / 36 (100.0%)	28 / 28 (100.0%)	36 / 36 (100.0%)	28 / 28 (100.0%)
Week 4	34 / 36 (94.4%)	26 / 28 (92.9%)	34 / 36 (94.4%)	26 / 28 (92.9%)
Week 8	35 / 36 (97.2%)	24 / 27 (88.9%)	35 / 36 (97.2%)	24 / 28 (85.7%)
Week 12	30 / 34 (88.2%)	21 / 23 (91.3%)	30 / 36 (83.3%)	21 / 28 (75.0%)
Week 16	29 / 30 (96.7%)	19 / 22 (86.4%)	29 / 36 (80.6%)	19 / 28 (67.9%)
Week 20	28 / 30 (93.3%)	19 / 20 (95.0%)	28 / 36 (77.8%)	19 / 28 (67.9%)
Week 24	27 / 29 (93.1%)	14 / 19 (73.7%)	27 / 36 (75.0%)	14 / 28 (50.0%)
Week 28	25 / 28 (89.3%)	16 / 16 (100.0%)	25 / 36 (69.4%)	16 / 28 (57.1%)
Week 32	19 / 27 (70.4%)	11 / 15 (73.3%)	19 / 36 (52.8%)	11 / 28 (39.3%)
	COMET-2			
	
	Cabozantinib	Mitoxantrone-Prednisone	Cabozantinib	Mitoxantrone-Prednisone

Baseline	53 / 53 (100.0%)	54 / 54 (100.0%)	53 / 53 (100.0%)	54 / 54 (100.0%)
Week 3	52 / 53 (98.1%)	52 / 54 (96.3%)	52 / 53 (98.1%)	52 / 54 (96.3%)
Week 6	46 / 52 (88.5%)	49 / 53 (92.5%)	46 / 53 (86.8%)	49 / 54 (90.7%)
Week 12	37 / 41 (90.2%)	39 / 42 (92.9%)	37 / 53 (69.8%)	39 / 54 (72.2%)
Week 18	25 / 27 (92.6%)	17 / 21 (81.0%)	25 / 53 (47.2%)	17 / 54 (31.5%)
Week 24	15 / 22 (68.2%)	12 / 13 (92.3%)	15 / 53 (28.3%)	12 / 54 (22.2%)

PRO-CTCAE: Patient-Reported Outcomes version of the Common Terminology Criteria for Adverse Events. “# Completed” refers to patients who completed the PRO-CTCAE assessment, “# Eligible for Completion” refers to patients who remained eligible to complete the PRO-CTCAE assessment, and “# Consented” refers to patients who consented to participate. The completion rate was calculated as the ratio of the number of patients with observed PRO-CTCAE scores to the number of patients eligible to complete the PRO-CTCAE assessment. The available data rate was calculated as the ratio of the number of patients with observed PRO-CTCAE scores to the total number of patients. The PRO-CTCAE was collected after Week 24 in COMET-2, but these time points were excluded from analysis due to fewer than 10 patients per arm still participating.

**Table 3.** Comparison of Analysis Strategies for Estimating Average Treatment Effects on the PRO-CTCAE at Week 12 in A091105

PRO-CTCAE Item	Sample Size		Average Treatment Effect (95% Confidence Interval)			
	MCAR-Based Method	MAR-Based Methods	Two-Sample *t*-Test	Mixed Model	Multiple Imputation No Auxiliary Variables	Multiple Imputation With Auxiliary Variables
Insomnia (S)	50	64	−0.250 (−0.829, 0.329)	−0.311 (−0.875, 0.252)	−0.277 (−0.840, 0.286)	—
Insomnia (I)	50	64	**−0.617 (−1.189, −0.044)**	−0.530 (−1.135, 0.075)	−0.487 (−1.061, 0.086)	—
Constipation (S)	50	64	−0.067 (−0.637, 0.503)	−0.121 (−0.630, 0.389)	−0.170 (−0.739, 0.399)	—
Pain (F)	50	64	−0.667 (−1.340, 0.007)	**−0.798 (−1.461, −0.135)**	**−0.738 (−1.381, −0.094)**	**−0.765 (−1.452, −0.079)**
Pain (S)	50	64	−0.483 (−1.078, 0.111)	−0.522 (−1.137, 0.093)	−0.491 (−1.114, 0.131)	−0.499 (−1.125, 0.127)
Pain (I)	51	64	−0.181 (−0.812, 0.450)	−0.213 (−0.860, 0.433)	−0.184 (−0.801, 0.433)	−0.162 (−0.792, 0.467)
Fatigue (S)	51	64	−0.300 (−0.972, 0.372)	−0.307 (−0.908, 0.295)	−0.255 (−0.911, 0.402)	−0.323 (−0.955, 0.309)
Fatigue (I)	51	64	−0.205 (−0.917, 0.508)	−0.160 (−0.811, 0.492)	−0.178 (−0.850, 0.494)	−0.167 (−0.841, 0.506)
Nausea (F)	51	64	0.081 (−0.418, 0.580)	0.160 (−0.311, 0.631)	0.143 (−0.379, 0.664)	0.101 (−0.408, 0.609)
Nausea (S)	51	64	0.095 (−0.407, 0.598)	0.165 (−0.301, 0.630)	0.158 (−0.395, 0.711)	0.133 (−0.390, 0.655)
Vomiting (F)	51	64	−0.105 (−0.379, 0.170)	−0.097 (−0.316, 0.122)	−0.131 (−0.433, 0.171)	−0.120 (−0.404, 0.163)
Vomiting (S)	51	64	−0.186 (−0.480, 0.109)	−0.174 (−0.425, 0.077)	−0.128 (−0.484, 0.227)	−0.196 (−0.495, 0.103)
Diarrhea (F)	51	64	0.014 (−0.639, 0.667)	0.327 (−0.291, 0.946)	0.279 (−0.469, 1.026)	0.371 (−0.421, 1.164)
Hand-Foot Syndrome (S)	51	64	**0.814 (0.304, 1.324)**	**0.841 (0.438, 1.244)**	**0.880 (0.353, 1.406)**	**0.833 (0.308, 1.357)**
Hand-Foot Syndrome (I)	51	64	**0.757 (0.259, 1.256)**	**0.812 (0.368, 1.256)**	**0.815 (0.269, 1.361)**	**0.762 (0.218, 1.307)**
Decreased Appetite (S)	51	64	−0.133 (−0.596, 0.330)	−0.220 (−0.673, 0.233)	−0.190 (−0.658, 0.279)	−0.244 (−0.697, 0.209)
Decreased Appetite (I)	51	64	−0.281 (−0.678, 0.116)	−0.339 (−0.725, 0.047)	−0.378 (−0.852, 0.097)	−0.385 (−0.850, 0.079)
Mouth/Throat Sores (S)	51	64	0.152 (−0.044, 0.348)	0.182 (−0.115, 0.479)	0.114 (−0.103, 0.332)	0.141 (−0.064, 0.346)
Rash (P)^1^	51	64	**10.857 (2.140, 55.081)**	**9.889 (2.213, 44.194)**	**7.318 (1.196, 44.775)**	**6.507 (1.069, 39.627)**

PRO-CTCAE: Patient-Reported Outcomes version of the Common Terminology Criteria for Adverse Events; CTCAE: Common Terminology Criteria for Adverse Events; (F): frequency; (S): severity; (I): interference; (P): presence; MCAR: missing completely at random; MAR: missing at random. The two-sample *t*-test is an MCAR-based method, whereas mixed modeling and multiple imputation (with and without CTCAE grades as auxiliary variables) are MAR-based methods. Significant results (*p* < 0.05) are in bold.

1 Odds ratios from a logistic regression model or generalized linear mixed model are reported for between-arm comparisons on patient-reported presence of rash. For this item, convergence issues precluded us from including PRO-CTCAE scores from all cycles (i.e., baseline through Week 32) in the mixed model. Thus, we only included PRO-CTCAE from a subset of cycles (i.e., baseline through Week 28) in the mixed model to achieve convergence.

**Table 4.** Comparison of Analysis Strategies for Estimating Average Treatment Effects on the PRO-CTCAE at Week 12 in COMET-2

PRO-CTCAE Item	Sample Size		Average Treatment Effect (95% Confidence Interval)			
	MCAR-Based Method	MAR-Based Methods	Two-Sample *t*-Test	Mixed Model	Multiple Imputation No Auxiliary Variables	Multiple Imputation With Auxiliary Variables
Insomnia (S)	76	107	0.216 (−0.278, 0.710)	0.203 (−0.233, 0.639)	0.118 (−0.344, 0.580)	0.148 (−0.331, 0.627)
Insomnia (I)	76	107	0.158 (−0.388, 0.704)	0.105 (−0.359, 0.569)	0.097 (−0.407, 0.601)	0.104 (−0.426, 0.635)
Constipation (S)	76	107	0.058 (−0.497, 0.613)	0.198 (−0.279, 0.674)	0.196 (−0.337, 0.728)	0.249 (−0.328, 0.827)
Pain (F)	76	107	−0.247 (−0.743, 0.250)	−0.379 (−0.767, 0.010)	−0.421 (−0.908, 0.066)	−0.305 (−0.821, 0.212)
Pain (S)	76	107	−0.349 (−0.779, 0.082)	**−0.374 (−0.723, −0.025)**	−0.386 (−0.825, 0.052)	−0.336 (−0.811, 0.138)
Pain (I)	76	107	−0.335 (−0.882, 0.211)	−0.369 (−0.831, 0.093)	−0.365 (−0.890, 0.160)	−0.276 (−0.839, 0.286)
Fatigue (S)	76	107	−0.019 (−0.492, 0.455)	−0.025 (−0.426, 0.376)	−0.069 (−0.529, 0.391)	−0.096 (−0.589, 0.396)
Fatigue (I)	76	107	−0.098 (−0.647, 0.450)	−0.130 (−0.593, 0.333)	−0.154 (−0.686, 0.377)	−0.215 (−0.782, 0.352)
Nausea (F)	76	107	0.239 (−0.276, 0.754)	0.266 (−0.236, 0.768)	0.251 (−0.266, 0.767)	0.270 (−0.226, 0.766)
Nausea (S)	76	107	0.367 (−0.124, 0.859)	0.345 (−0.127, 0.818)	0.351 (−0.152, 0.853)	0.382 (−0.099, 0.863)
Vomiting (F)	76	107	0.078 (−0.316, 0.471)	0.125 (−0.242, 0.493)	0.190 (−0.200, 0.579)	0.178 (−0.210, 0.566)
Vomiting (S)	76	107	0.129 (−0.256, 0.514)	0.167 (−0.231, 0.566)	0.168 (−0.213, 0.549)	0.165 (−0.220, 0.551)
Diarrhea (F)	76	107	**0.640 (0.092, 1.188)**	**0.624 (0.174, 1.074)**	**0.657 (0.124, 1.190)**	**0.603 (0.060, 1.147)**
Decreased Appetite (S)	76	107	0.522 (−0.023, 1.067)	0.453 (−0.015, 0.921)	0.431 (−0.147, 1.009)	0.453 (−0.097, 1.003)
Decreased Appetite (I)	76	107	0.311 (−0.209, 0.830)	0.230 (−0.256, 0.716)	0.162 (−0.392, 0.716)	0.176 (−0.348, 0.701)
Numbness/Tingling (S)	76	107	0.455 (−0.048, 0.958)	0.324 (−0.111, 0.759)	0.330 (−0.142, 0.802)	0.356 (−0.119, 0.831)
Numbness/Tingling (I)	76	107	0.405 (−0.075, 0.884)	0.307 (−0.110, 0.725)	0.270 (−0.183, 0.724)	0.273 (−0.181, 0.727)
Mouth/Throat Sores (S)	76	107	0.130 (−0.233, 0.493)	0.121 (−0.189, 0.431)	0.103 (−0.286, 0.492)	0.115 (−0.243, 0.474)
Shortness of Breath (S)	75	107	−0.238 (−0.737, 0.260)	−0.108 (−0.545, 0.329)	−0.075 (−0.579, 0.428)	−0.094 (−0.583, 0.395)
Shortness of Breath (I)	75	107	−0.107 (−0.656, 0.441)	0.051 (−0.417, 0.518)	0.087 (−0.483, 0.657)	0.063 (−0.500, 0.627)
Rash (P)^1^	76	107	0.859 (0.238, 3.099)	0.749 (0.199, 2.813)	0.915 (0.278, 3.016)	1.006 (0.293, 3.457)

PRO-CTCAE: Patient-Reported Outcomes version of the Common Terminology Criteria for Adverse Events; CTCAE: Common Terminology Criteria for Adverse Events; (F): frequency; (S): severity; (I): interference; (P): presence; MCAR: missing completely at random; MAR: missing at random. The two-sample *t*-test is an MCAR-based method, whereas mixed modeling and multiple imputation (with and without CTCAE grades as auxiliary variables) are MAR-based methods. Significant results (*p* < 0.05) are in bold.

1 Odds ratios from a logistic regression model or generalized linear mixed model are reported for between-arm comparisons on patient-reported presence of rash.

**Table 5.** CTCAE Grades at Week 12

CTCAE Term	CTCAE Grade					
	0	1	2	3	4	5
A091105						

Fatigue	40	21	2	1	—	—
Papulopustular Rash	54	10	—	—	—	—
Palmar-Plantar Erythrodysesthesia Syndrome	46	15	3	—	—	—
Diarrhea	54	8	1	1	—	—
Anorexia	55	7	2	—	—	—
Nausea	49	13	2	—	—	—
Vomiting	60	3	1	—	—	—
Abdominal Pain	58	3	2	1	—	—
Mucositis Oral	61	3	—	—	—	—
Arthralgia	57	7	—	—	—	—
Myalgia	60	4	—	—	—	—

COMET-2						

Insomnia	97	6	3	1	—	—
Constipation	79	16	10	2	—	—
Back Pain	93	2	7	5	—	—
Pain in Extremity	97	4	5	1	—	—
Arthralgia	96	5	6	—	—	—
Fatigue	69	10	24	4	—	—
Nausea	77	17	11	2	—	—
Vomiting	93	10	3	1	—	—
Diarrhea	89	13	5	—	—	—
Rash	103	3	1	—	—	—
Decreased Appetite	79	16	10	2	—	—
Peripheral Neuropathy	96	5	6	—	—	—
Mucositis Oral	98	6	2	1	—	—
Dyspnea	92	12	2	1	—	—

CTCAE: Common Terminology Criteria for Adverse Events.

## Plain English Summary

Safety and treatment tolerability have historically been assessed solely by clinicians via the Common Terminology Criteria for Adverse Events (CTCAE). However, clinician reports under-detect symptoms relative to patient reports. Therefore, the National Cancer Institute contracted development of the Patient-Reported Outcomes version of the CTCAE (PRO-CTCAE) item library, which is now widely implemented in cancer clinical trials. However, missing PRO-CTCAE responses from patients complicate reporting and analysis. As with other patient-completed questionnaires, patients with and without missing PRO-CTCAE responses may systematically differ, thus jeopardizing the validity and generalizability of clinical trial results. For example, patients with more severe symptoms might miss PRO-CTCAE assessments more often than patients with less severe symptoms, potentially leading to invalid conclusions being drawn from analyses based on only the remaining patients’ PRO-CTCAE responses. This paper focuses on analysis of the PRO-CTCAE when some patients’ responses are missing (Alliance A151912), with application to 2 randomized, double-blind, placebo-controlled, phase III trials: Alliance A091105 and COMET-2. In each trial, we applied various methods for comparing patient-reported symptoms across treatment arms while addressing missing PRO-CTCAE responses. We found that optimal methods use patients’ responses to the PRO-CTCAE at other time points to provide information about their would-be responses to the PRO-CTCAE at the time point of interest. Clinicians’ CTCAE grades did not provide useful information about patients’ missing PRO-CTCAE responses.

## Footnotes

1 The PRO-CTCAE does not include an item on interference of hand-foot syndrome with usual or daily activities. However, this item was written to mimic other PRO-CTCAE items.

2 In A091105, we conducted between-arm comparisons on the PRO-CTCAE at Week 12 because Week 12 was a time point of interest for evaluating patient-reported pain palliation. In COMET-2, we conducted between-arm comparisons on the PRO-CTCAE at Week 12 because the primary endpoint was pain response at Week 6 confirmed at Week 12. However, the analyses and results presented here are intended to illustrate different missing data strategies and not to replace results reported in other A091105 and COMET-2 publications.
