# Correction to: Missing data strategies for the Patient-Reported Outcomes version of the Common Terminology Criteria for Adverse Events (PRO-CTCAE) in Alliance A091105 and COMET-2

**DOI:** 10.1007/s11136-021-03000-2

**Published:** 2021-10-11

**Authors:** Gina L. Mazza, Molly M. Petersen, Brenda Ginos, Blake T. Langlais, Narre Heon, Mrinal M. Gounder, Michelle R. Mahoney, Alexander J. Zoroufy, Gary K. Schwartz, Lauren J. Rogak, Gita Thanarajasingam, Ethan Basch, Amylou C. Dueck

**Affiliations:** 1 Alliance Statistics and Data Center, Mayo Clinic, 13400 East Shea Boulevard, Scottsdale, AZ 85259 USA; 2 Department of Quantitative Health Sciences, Mayo Clinic, Scottsdale, AZ USA; 3 Memorial Sloan Kettering Cancer Center, New York, NY USA; 4 IQVIA Institute for Human Data Science, Raleigh-Durham, NC USA; 5 Alliance Statistics and Data Center, Mayo Clinic, Rochester, MN USA; 6 Department of Quantitative Health Sciences, Mayo Clinic, Rochester, MN USA; 7 Herbert Irving Comprehensive Cancer Center, Columbia University, New York, NY USA; 8 Division of Hematology, Mayo Clinic, Rochester, MN USA; 9 UNC Lineberger Comprehensive Cancer Center, University of North Carolina, Chapel Hill, NC USA

## Correction to: Quality of Life Research (2021) https://doi.org/10.1007/s11136-021-02968-1

The article “Missing data strategies for the Patient-Reported Outcomes version of the Common Terminology Criteria for Adverse Events (PRO-CTCAE) in Alliance A091105 and COMET-2”, written by Gina L. Mazza, Molly M. Petersen, Brenda Ginos, Blake T. Langlais, Narre Heon, Mrinal M. Gounder, Michelle R. Mahoney, Alexander J. Zoroufy, Gary K. Schwartz, Lauren J. Rogak, Gita Thanarajasingam, Ethan Basch and Amylou C. Dueck, was originally published electronically on the publisher’s internet portal on 21 August 2021 without open access. With the author(s)’ decision to opt for Open Choice the copyright of the article changed on 16 September 2021 to © The Author(s) 2021 and the article is forthwith distributed under a Creative Commons Attribution 4.0 International License, which permits use, sharing, adaptation, distribution and reproduction in any medium or format, as long as you give appropriate credit to the original author(s) and the source, provide a link to the Creative Commons licence, and indicate if changes were made. The images or other third party material in this article are included in the article’s Creative Commons licence, unless indicated otherwise in a credit line to the material. If material is not included in the article’s Creative Commons licence and your intended use is not permitted by statutory regulation or exceeds the permitted use, you will need to obtain permission directly from the copyright holder. To view a copy of this licence, visit http://creativecommons.org/licenses/by/4.0.

The original article has been corrected.

